# Salvage endovascular embolization of the left pulmonary artery for recurrent pseudoaneurysm

**DOI:** 10.1186/s40792-021-01306-4

**Published:** 2021-09-27

**Authors:** Yojiro Yutaka, Junichi Tasaki, Itsuki Yuasa, Kotaro Murakami, Hiroshi Date

**Affiliations:** 1 Department of Thoracic Surgery, Kyoto University Hospital, 54 Kawaharacho, Shogoin, Sakyo-ku, Kyoto, 606-8507 Japan; 2 Department of Cardiovascular Medicine, Kyoto University Hospital, 54 Kawaharacho, Shogoin, Sakyo-ku, Kyoto, 606-8507 Japan

**Keywords:** Pulmonary pseudoaneurysm, Endovascular coiling, Pneumonectomy, Hemoptysis

## Abstract

**Background:**

Pulmonary pseudoaneurysm (PPA) is a potentially lethal complication of lung resection with a high risk of recurrence after endovascular coiling.

**Case presentation:**

We report a case in which recurrent hemoptysis due to PPA after left lower lobe sleeve resection was treated by endovascular embolization of the left main pulmonary artery as a salvage treatment. The first hemoptysis was managed by endovascular coil embolization with extracorporeal membrane oxygenation, but refractory hemorrhage occurred 3 months later due to penetration of the endovascular coil into the bronchial anastomosis site. Because left completion pneumonectomy was considered too high risk, the left main pulmonary artery was palliatively embolized using an Amplatzer vascular plug (St. Jude Medical, MN, USA) to totally disrupt the left pulmonary arterial flow.

**Conclusions:**

Total embolization of the left main pulmonary artery for repeated PPA rupture may be useful as a palliative treatment in patients unable to tolerate pneumonectomy.

## Background

Bronchovascular fistula occurs in 1–3% of patients during the first 4 weeks after a bronchoplastic procedure [1, 2]. Hemoptysis due to a bronchial fistula with a pulmonary pseudoaneurysm (PPA) is an extremely rare condition that requires emergency lifesaving treatment [3]. We report a case in which recurrent hemoptysis in a patient with a PPA following left lower lobe sleeve resection after induction chemoradiation therapy (ICRT) was treated endovascularly as an alternative to pneumonectomy.

## Case presentation

**Fig. 1:**
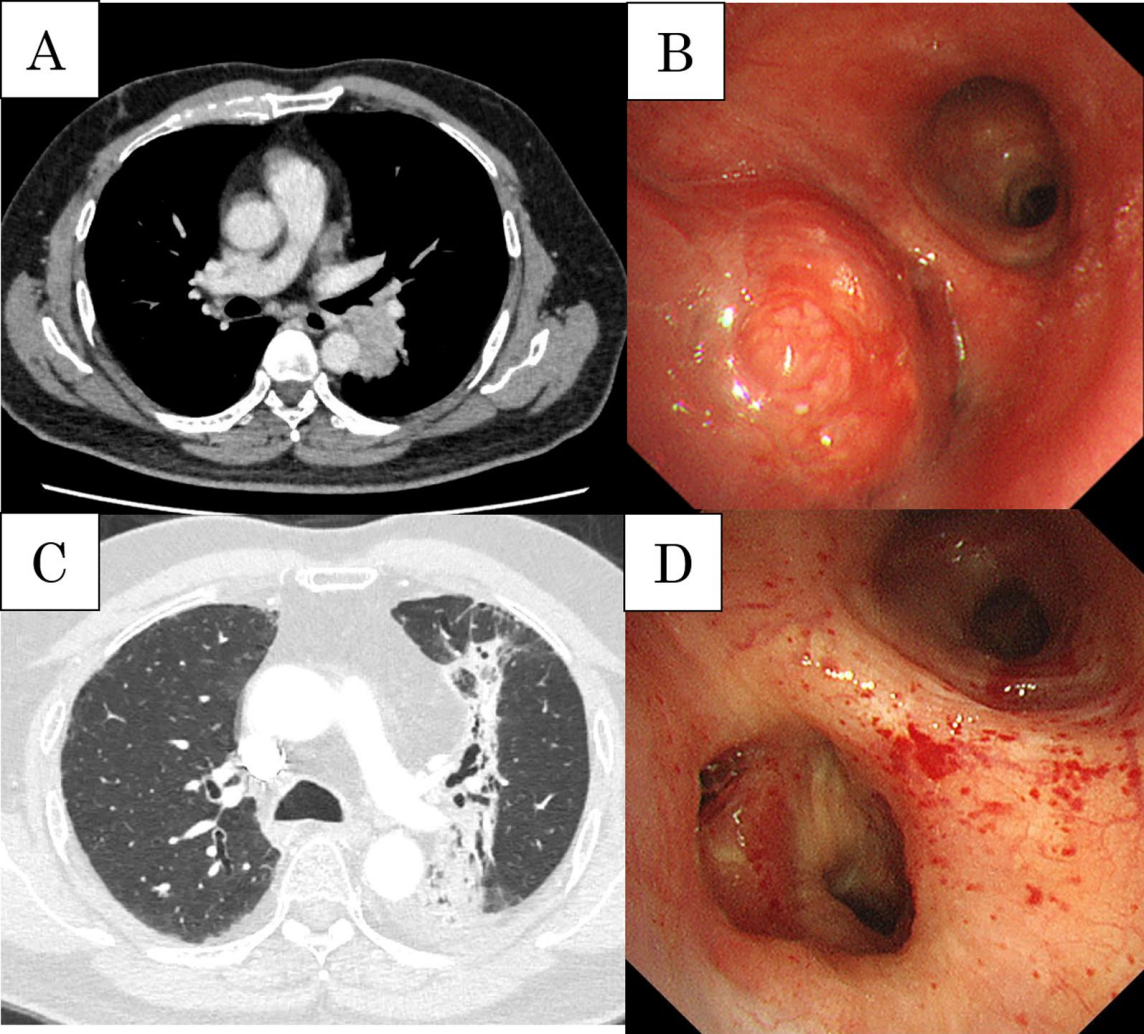
Preoperative CT and bronchoscopic findings. **A** Initial CT showing squamous cancer in the left lower lung lobe. **B** The tumor protruding from the left lower bronchus and invading the left main bronchus. **C** CT showing moderate radiation pneumonitis after induction chemoradiation therapy. **D** Tumor regression after preoperative therapy shown by the appearance of the left lower bronchus

**Fig. 2:**
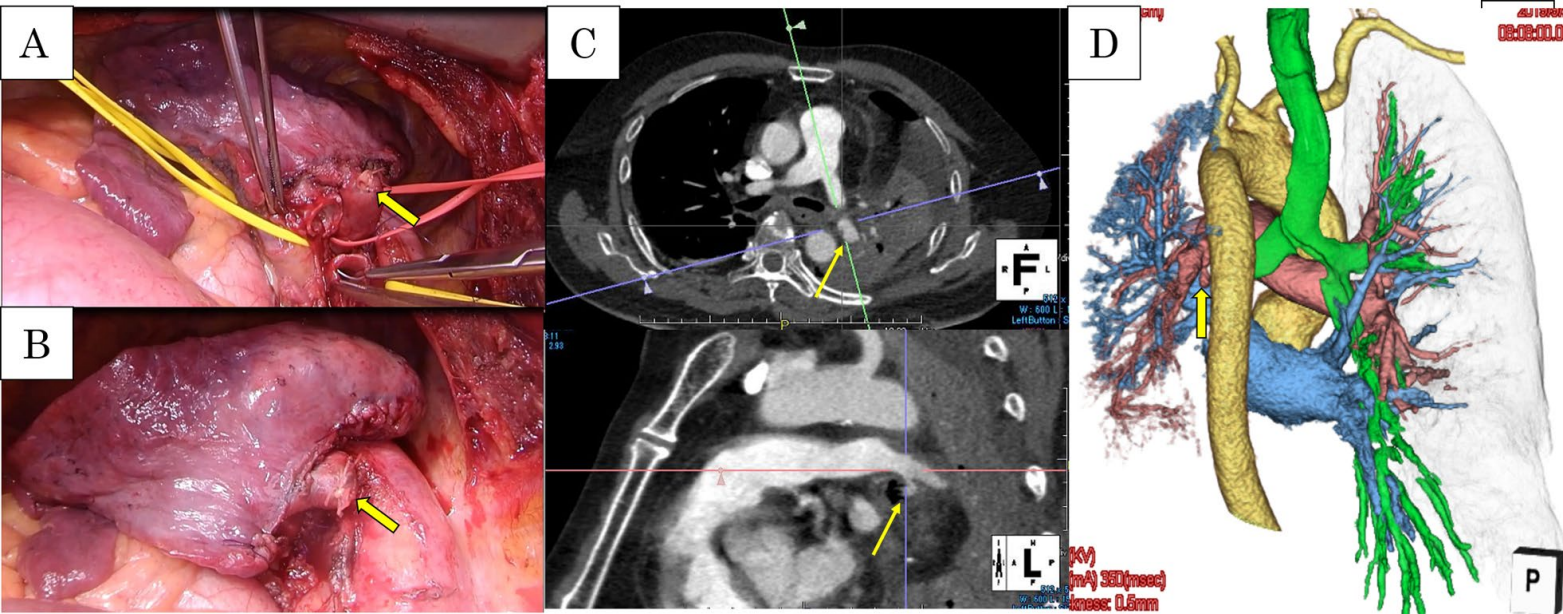
Intraoperative findings. **A**, **B** Positional relationship between the bronchial anastomosis site and A6 stump (yellow circle). **C**, **D** Position of the pulmonary pseudoaneurysmal fistula. The yellow arrow indicates the pulmonary pseudoaneurysm

**Fig. 3:**
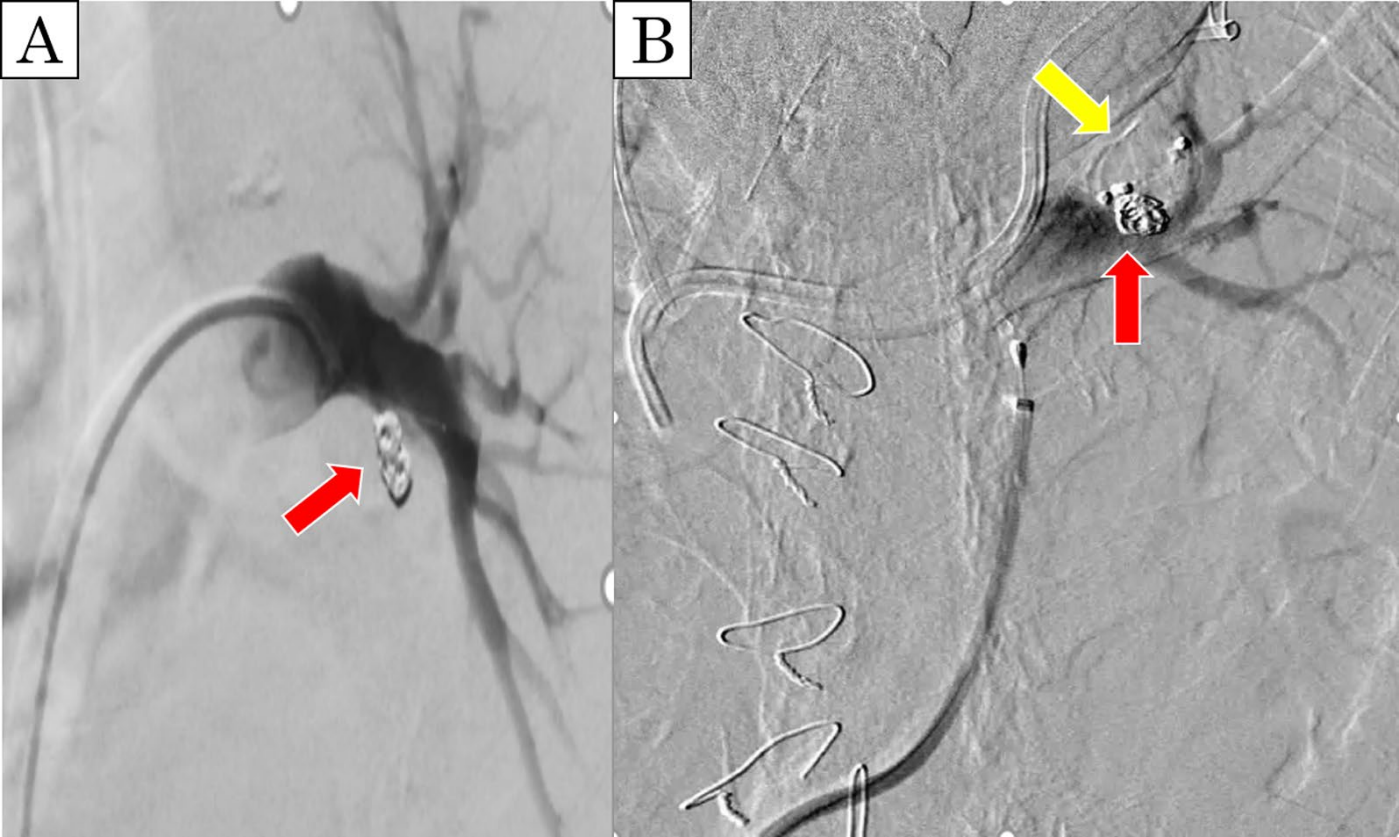
Angiography findings. **A** Angiography showing the pulmonary aneurysm treated by emergency endovascular coil embolization with the use of *N*-butyl cyanoacrylate (red arrow indicates a coil). **B** Angiography performed immediately after central embolization of the left main pulmonary artery using an Amplatzer vascular plug (St. Jude Medical, MN, USA) (yellow arrow indicates a vascular plug)

**Fig. 4:**
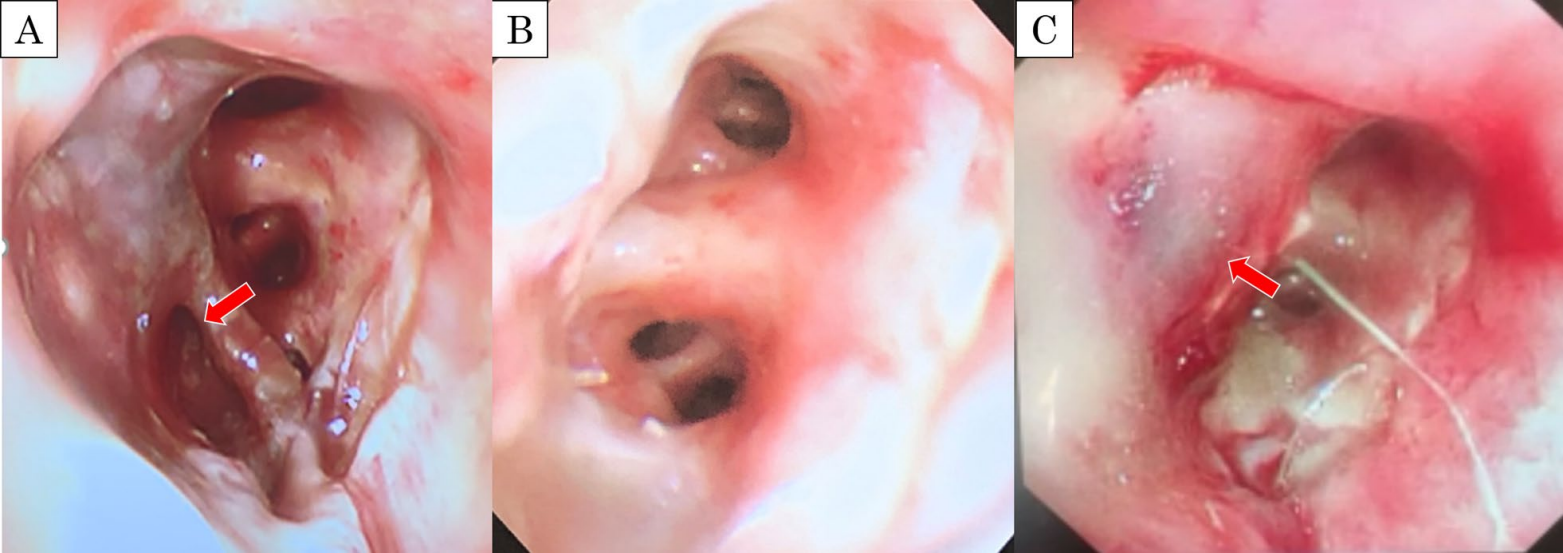
Serial bronchoscopic findings. **A** The half circle of the bronchial anastomosis site indicates ischemia on postoperative day (POD) 17 (red arrow). **B** The anastomosis site undergoing epithelialization on POD 34. **C** The pulmonary artery is stuck in the bronchial anastomosis site on POD 114, suggesting impending rupture (red arrow)

The patient was a 60-year-old man with cT2bN2M0 (Sq, 45 mm, LN#4L) (Fig. 1A). Bronchoscopy showed a tumor protruding from the left lower bronchus and invading the left main bronchus (Fig. 1B); however, a tissue biopsy from the second carina was negative. Pulmonary function testing prior to ICRT revealed that the vital capacity (VC) was 3330 ml (%VC: 82.4%), forced expiratory volume in 1 s (FEV1) was 2642 ml (%FEV1: 80.2%), and % carbon monoxide diffusing capacity of the lung (%DLCO) was 64.2%. After ICRT (carboplatin/paclitaxel + 40 Gy), he developed moderate radiation pneumonitis and ipsilateral pulmonary artery emboli from deep venous thrombosis, which required medical treatment with oral steroids and a novel oral anticoagulant drug (Fig. 1C). Re-evaluation of bronchoscopy and positron emission tomography–computed tomography suggested disappearance of FDG uptake by lymph node #4L (Fig. 1D). Although the preoperative pulmonary function could not be adequately evaluated due to the severe cough caused by radiation pneumonitis, he was considered unable to tolerate pneumonectomy because the 6-min walk test revealed marked desaturation on exercise (lowest oxygen saturation was 85%). Therefore, he was scheduled to undergo left lower lobe sleeve resection rather than left pneumonectomy. Left lower sleeve resection required pulmonary arterioplasty of A6 with side-clamping. After dissection of mediastinal lymph nodes #4L and #7, bronchial anastomosis was performed with 4-0 polydioxanone, and a pedicled pericardial fat pad was interposed between the pulmonary artery and bronchial anastomosis (Fig. 2A, B). Pathological examination revealed complete remission (Ef3). The postoperative course was uneventful. Heparinization was restarted plus 5 mg of oral steroids on postoperative day (POD) 1; heparin was replaced by an oral anticoagulant after drain removal on POD 12. Although discharge was planned on POD 17, sudden massive hemoptysis occurred, requiring emergency one-lung intubation with extracorporeal membrane oxygenation (ECMO) and muscle relaxants. Enhanced CT revealed a PPA (Fig. 2C, D) and severe right lung damage due to inhalation of a considerable amount of hemoptysis; as surgical intervention including left completion pneumonectomy was considered high risk, emergency endovascular coil embolization with the use of *N*-butyl cyanoacrylate was conducted in the sutured A6 branch (Fig. 3A). Bronchoscopy revealed massive endobronchial occlusion due to thrombi, and ischemic changes and dehiscence of half of the anastomosis site in the left bronchus (Fig. 4A). Daily endobronchial toileting resulted in gradual improvement of oxygenation, which enabled the removal of ECMO on POD 34. The bronchial anastomosis site gradually regenerated conservatively (Fig. 4B), but hemoptysis recurred on POD 114. Bronchoscopic examination under ECMO demonstrated that the endovascular coils had partially migrated into the left main bronchus and deteriorated the bronchial healing. The pulsatile pulmonary artery was stuck in part of the bronchial anastomosis site, which suggested impending rupture (Fig. 4C). We planned a completion pneumonectomy; however, the patient was deemed unable to tolerate pneumonectomy because the considerable amount of hemoptysis had caused further deterioration of the remaining lung, and the ECMO had caused thrombocytopenia and acute renal failure requiring continuous hemodiafiltration. As an alternative to pneumonectomy, palliative endovascular embolization of the left main pulmonary artery was conducted using an Amplatzer vascular plug (St. Jude Medical, MN, USA) to totally disrupt the left pulmonary arterial flow (Fig. 3B). Although refractory pneumonia with empyema due to insufficient intrapulmonary circulation occurred, this treatment prevented hemoptysis recurrence and enabled withdrawal from ECMO and continuous hemodiafiltration. The patient was discharged to home without oxygen inhalation on POD 359. No late complications due to left pulmonary embolization were seen at 1 year after discharge. His current lung function tests revealed a VC of 1570 ml (%VC: 39.0%), FEV1 of 1360 ml (%FEV1: 41.6%), and %DLCO of 26.7%.

## Discussion

PPA following lung resection is associated with high mortality. As many as 50% of PPA are diagnosed postmortem, as there are no apparent clinical signs until massive lethal hemoptysis [3, 4]. PPA most commonly occurs after iatrogenic trauma, chest trauma, or pulmonary infection [4]. In our case, a fistula developed between the bronchial anastomosis site and the PPA, possibly caused by poor bronchial healing due to devascularization from lymph node dissection in pulmonary fibrosis and intimal damage from the clamping of the pulmonary artery. Buttressing of the anastomosis site using omentum, muscle flap, and pericardium reportedly helps preserve the blood supply and prevent bronchovascular fistula [5]; however, it is crucial to completely isolate the anastomosis site from the airway.

Radiation pneumonitis is a serious adverse event after thoracic radiotherapy. Sugimoto et al. [6] reported that radiation pneumonitis may contribute to the development of a destroyed lung after lung resection, as lungs with radiation pneumonitis do not have normal wound healing mechanisms and are susceptible to infection. Furthermore, a recent prospective study showed that extended lung resection after concurrent chemotherapy and high-dose radiation (60 Gy) is associated with increased 30- and 90-day mortality rates compared with anatomic lung resection after concurrent chemotherapy and high-dose radiation [7]. Special attention should be paid to bronchial healing, because of the bronchial blood flow impairment caused by chemotherapy and radiation therapy.

Surgical treatment for bronchovascular fistula comprises fistula resection with reconstruction of the pulmonary artery or bronchus, or simply pneumonectomy. However, the morbidity and mortality of this emergency procedure is extremely high, and its recommendation depends on the balance between underlying conditions and expected complications. Endovascular coiling effectively achieves temporary hemostasis [8]; however, for PPA, rebleeding occurs in approximately 12.5% of patients due to the lack of adventitial wall in the pulmonary artery [9]. Therefore, surgeons should consider surgical intervention if technically possible and tolerable to the patient. In the present case, considering that the blood flow in the left pulmonary artery was eventually almost eliminated and the cardiopulmonary load was probably similar to that after pneumonectomy, left completion pneumonectomy might have been possible on POD 34 when the ECMO was removed.

**Fig. 5:**
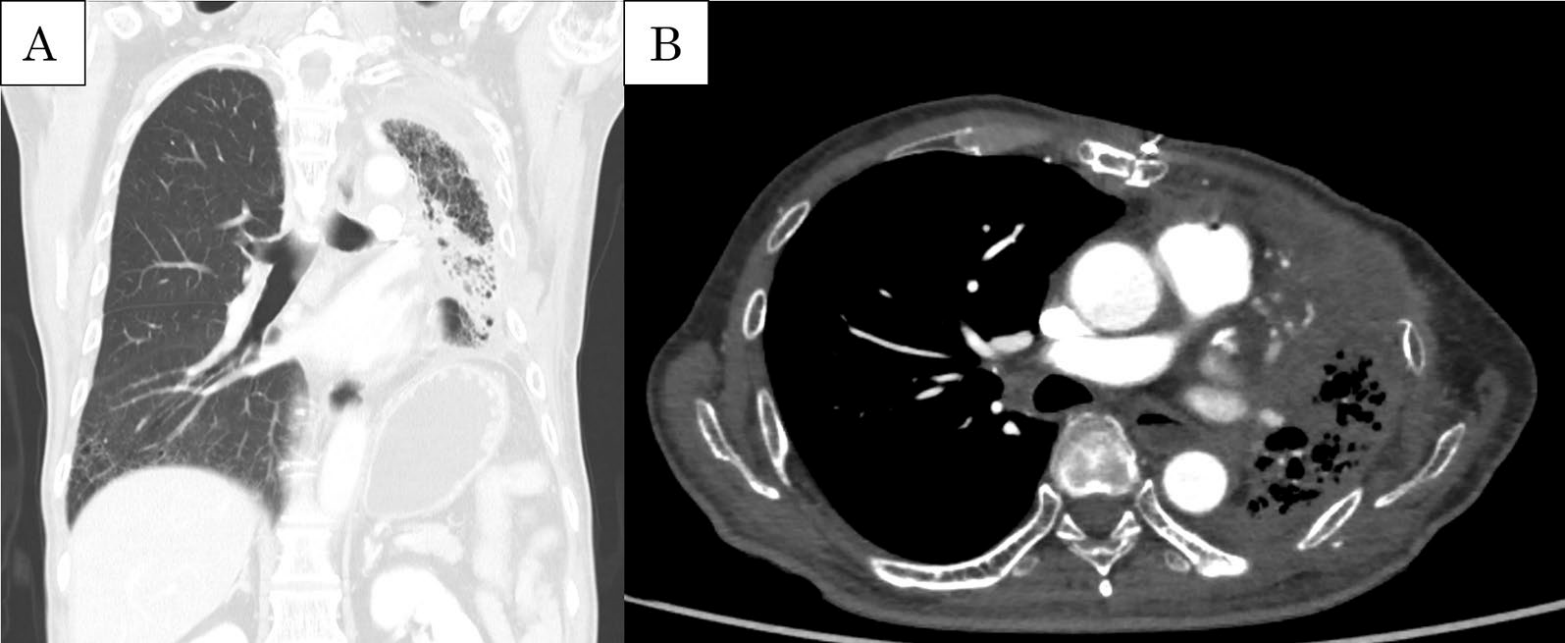
Current CT findings. **A** Coronal section showing moderate fibrosis in the left lung. **B** Enhanced CT showing the left pulmonary blood flow supplied by the bronchial artery and collateral circulation from the chest wall

The long-term effect of embolization of a central pulmonary artery to cut off blood supply to an entire lung has not been reported. Therefore, our concerns before performing total pulmonary embolization included possible pulmonary hypertension, pulmonary infection due to insufficient pulmonary circulation, and exacerbation of bronchial anastomotic ischemia. However, as the left lung was already severely damaged by repeated hemoptysis and had decreased respiratory function, the risk of respiratory failure due to imbalanced ventilation and blood flow after embolization was considered minimal [10]. Regarding the bronchial blood supply, there are some reports that pulmonary sequestration totally supplied by an anomalous systemic artery supply has been successfully treated using a vascular plug, without causing severe long-term complications [11]. The low incidence of pulmonary infarction and lack of ischemic complications after embolization are likely explained by the available collateral circulation from the bronchial, intercostal, inferior phrenic, and other nearby arteries (Fig. 5). The vascular plug gradually occluded the pulmonary artery by promoting clot formation, which might have enable the patient to adapt to the gradual hemodynamic change. Our patient has not required readmission for the treatment of late pulmonary complications. However, the theoretical validity of this treatment should be carefully evaluated.

## Conclusions

We presented a case of rescue from recurrent hemoptysis after endovascular coiling of PPA, which was managed by total embolization of the left pulmonary artery using a vascular plug. Although total embolization of the pulmonary artery cured the present patient, surgeons should be aware of the risks of endovascular coil migration to a fistula between the PPA and bronchial anastomosis site and possible late complications of total pulmonary embolization.

## Data Availability

All data generated or analyzed during this study are included in this published article.

## Abbreviations

PPA: Pulmonary pseudoaneurysm
ICRT: Induction chemoradiation therapy
VC: Vital capacity
FEV1: Forced expiratory volume in 1 s
DLCO: Carbon monoxide diffusing capacity of the lung
POD: Postoperative day
ECMO: Extracorporeal membrane oxygenation
